# Supplementary material for: Tree-based machine learning performed in-memory with memristive analog CAM
Source: Nat Commun. 2021 Oct 4;12:5806. doi: 10.1038/s41467-021-25873-0 (PMC8490381; doi:10.1038/s41467-021-25873-0)
Supplement: Supplementary file 1 — Supplementary information [file 41467_2021_25873_MOESM1_ESM.pdf]

Supplementary Information for Tree-based machine learning performed in-memory with memristive analog CAM

Giacomo Pedretti[1,*], Catherine E. Graves[1,*], Sergey Serebryakov[1], Ruibin Mao[2], Xia Sheng[1], Martin Foltin[1], Can Li[1,2], and John Paul Strachan[3,4,*]

[1]Hewlett Packard Labs, Milpitas (CA), USA
[2]The University of Hong Kong, Hong Kong SAR, China
[3]Peter Grünberg Institut (PGI-14), Forschungszentrum Jülich GmbH, Jülich, Germany
[4]RWTH Aachen University, Aachen, Germany
[*]giacomo.pedretti@hpe.com, catherine.graves@hpe.com, j.strachan@fz-juelich.de

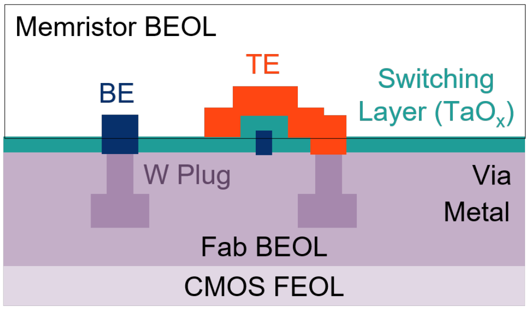

Supplementary Figure 1. Memristor stack. Cross-section of a memristor integrated on a CMOS chip. NMOS fabrication is carried out in a foundry with 180 nm technology. Ta/TaOx/Pt thin film stack is integrated aligning to the exposed metals by lab memristor BEOL process.

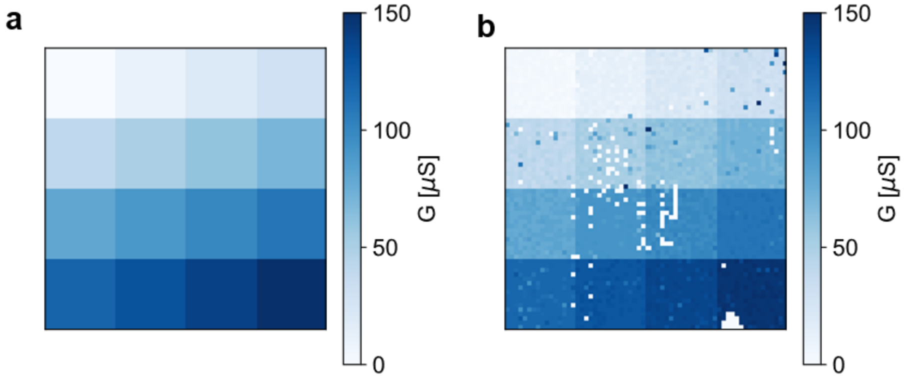

Supplementary Figure 2. Memristor programming. (a) Target 64 × 64 matrix divided in 16 different box, each comprising 256 elements of different values. (b) Results of the measured programmed matrix. An integrated circuit[1] comprising 3 64 × 64 memristor arrays, sensing and routing circuitry was used to study the noise in programming each level. Some devices were stuck in a low (white) or high conductance (strong blue) that can not be recovered. For this reason we considered only the best 128 out of 256 devices for the cumulative distribution plots.

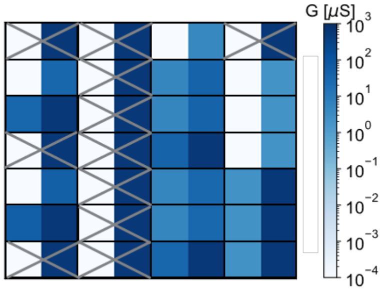

Supplementary Figure 3. Programmed conductance in a analog CAM arrays for the classification of Iris dataset, ’X’ corresponds to ’don’t care’ namely memristor programmed in the wider possible range with M1 = HRS and M2 = LRS

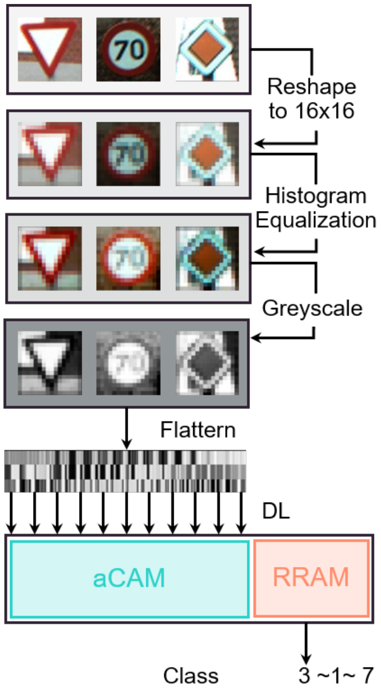

Supplementary Figure 4. Random forest training pre processing. The same procedure of the reference benchmark[2] was applied. KUL traffic sing data set consist of 32×32 256 levels RGB image that were reshaped to 16×16. After that an histogram equalization technique was applied and finally the image was converted to grey scale. After flattening to a 1×256 array, the image is applied to analog CAM DL and the matched class activate the corresponding memristors on the RAM array for majority voting.

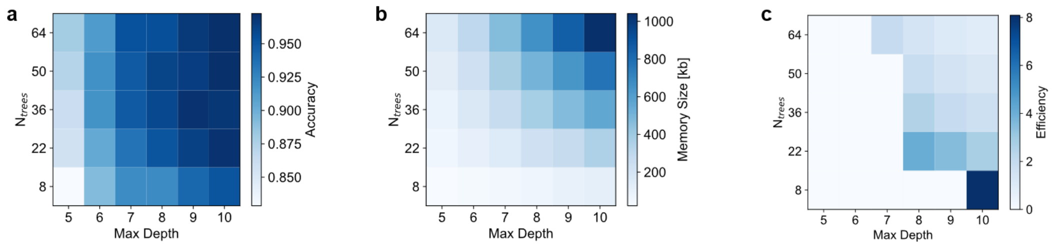

Supplementary Figure 5. Random Forest hyperparameters tuning. (a) Accuracy as function of number of tree N_{trees} and maximum depth of each tree. (b) Memory size with 32×32 tiles as function of N_{trees} and maximum depth of each tree. (c) Efficiency namely $Efficiency = Accuracy/MemorySize$ as function of N_{trees} and maximum depth of each tree. Points with accuracy < 0.96 were excluded to be sure of matching the reference 0.94 accuracy[2] once all non idealities are included.

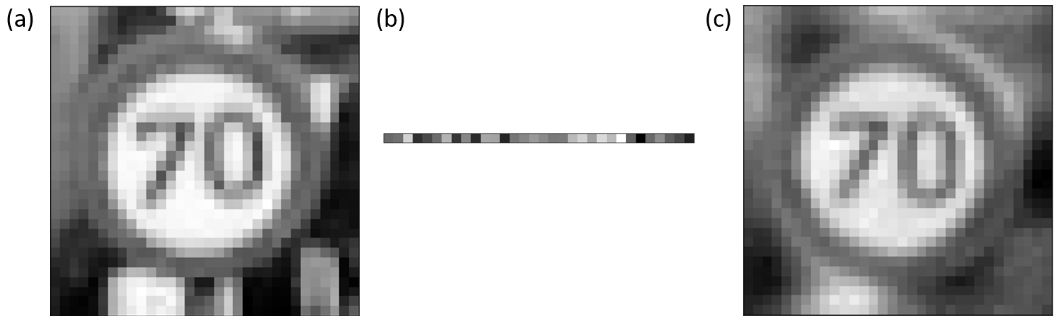

Supplementary Figure 6. Example of autoencoder compression and reconstruction effect. (a) A 32×32 image is fed into the autoencoder encode unit resulting in its 32×1 latent representation (b). The latent representation is used as feature vector for training a RF. Alternatively, by applying the latent representation as input to the decode unit a 32×32 reconstructed image can be obtained.

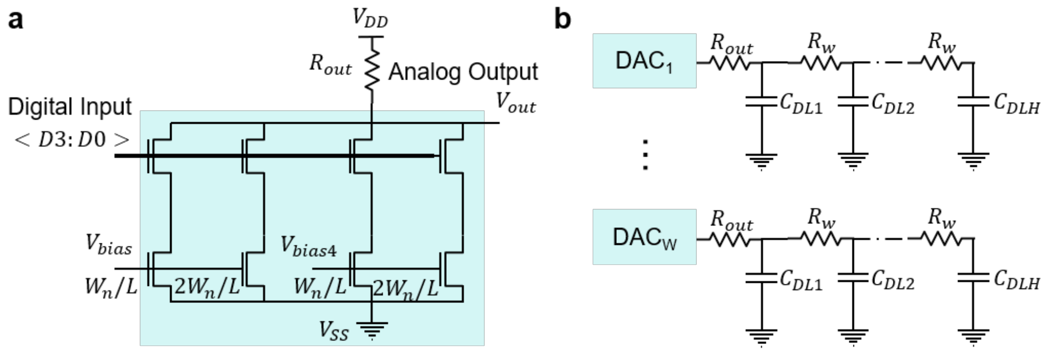

Supplementary Figure 7. Digital to analog converter (DAC) design and DL power consumption calculation. (a) Schematic of the current steering DAC considered for this work[3]. R_{out} was optimized by considering the clock time and parasitic resistance and conductance due to wires. (b) Schematic of a DL circuit where each analog CAM has a parasitic capacitance C_{DL} and analog CAM cells are connected with each other with a wire of resistance R_w. Elmore's theorem was considered to compute the propagation delay namely $\tau = \sum_{i=0}^{H}(R_{out} + iR_w)C_{DL} = C_{DL}\left[R_{out}H + R_w\frac{H(H-1)}{2}\right]$, with H height of the analog CAM tile, or number of analog CAM cells in a column.

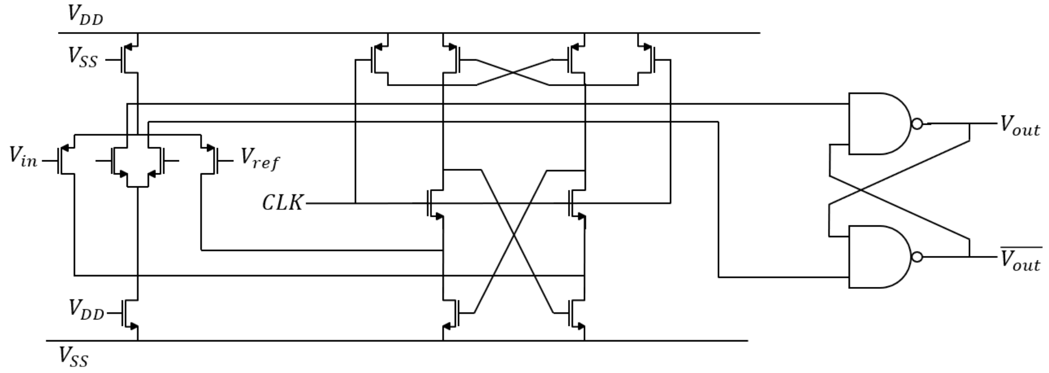

Supplementary Figure 8. Sense amplifier (SA) circuit schematic. V_{in} divides the current of the input differential stage into the second stage unevenly. The balance causes the second stage to snap (either to V_{DD} or V_{SS} when the clocks are activated. The NADD gates at the output create a latch function of hold the value.

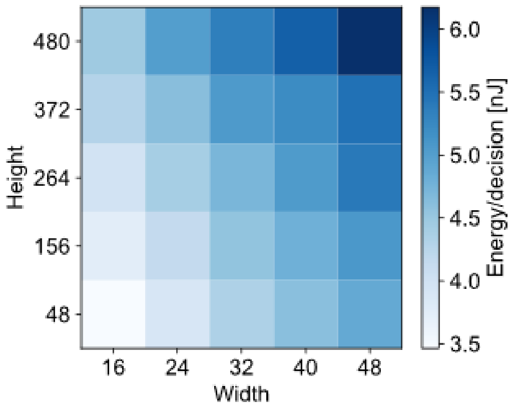

Supplementary Figure 9. Energy per decision as function of height (H) and width (W) of each analog CAM tile. It is possible to see that the trend is opposite compared to the energy per decision per node which is due to the optimal pipeline (i.e. continuous input data flow) considered.

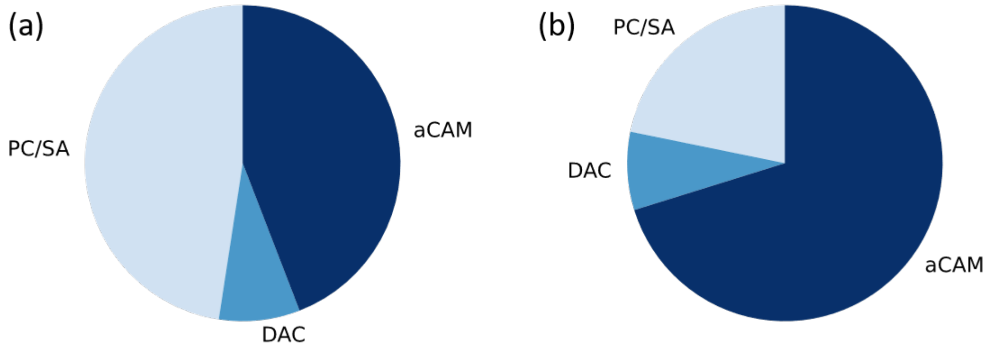

Supplementary Figure 10. Breakdown of power consumption for different components, namely pre-charge and sense amplifiers (PC/SA), digital to analog converters (DAC), and analogCAM arrays (aCAM) for 180 nm (a) and 65 nm (b) technology node. By scaling the technology node the contribute in the overall power of analog CAM cells becomes dominant due to the conductance range which stays approximately fixed.Analog CAM cell transistor are scaled down with the technology node, but T1 and T3 are oversized to allow enough current for programming the memristor device to low conductance. In this case, the array dimensions are kept constant with $H = 480$ and $W = 16$.

1 Supplementary Note 1: Compact model details

Data collected from the post-tape out simulations were modeled with the following equations:

- Sub-threshold regime $I_{D,T1} = I_{D0}\exp\left(\frac{V_{DL}}{\alpha}\right)$
- Intermediate regime $I_{D,T1} = I'_{D0}\exp\left(\frac{V_{DL}}{\alpha}\right)$
- Approximate ohmic regime $I_{D,T1} = k_1(V_{DL} - V_{Th,T1})$
- Saturation regime $I_{D,T2} = k_2(V_{DS,T1} - V_{Th,T2})^2$
- Inverter behavioural model $V_{G,T6} = \frac{-0.8}{1+\exp(-\beta(V_{GS,T4}+\gamma))} + 0.8$

Fig. 11 shows the comparison between the model equations and collected data demonstrating a good agreement. Note that this is a behavioral model thus equations were simplify to speed up simulations of large scale arrays. Model fitting parameters are shown in Supplementary Table 1

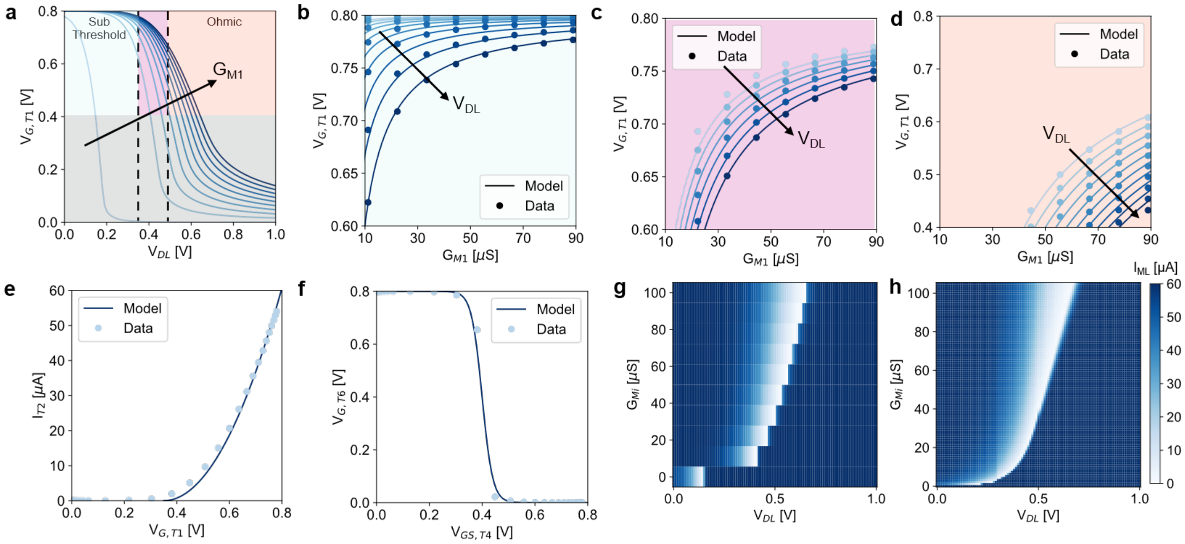

Supplementary Figure 11. Analog CAM model details. (a) Circuit simulations results $V_{G,T1}$ as function of V_{DL} for different programmed G_{M1} evidencing two main working region namely sub threshold for $V_{DL} < 0.3$ (light blue) and ohmic (or linear) for V_{DL}>0.5 (orange). We also considered an intermediate subthreshold region for $0.3 < V_{DL} < 0.5$ (purple) with subtreshold conduction but slightly different fitting parameters. Circuit simulation results and model calculation of $V_{G,T1}$ as function of G_{M1} in the subthreshold regime (b), intermediate regime (c), and approximate ohmic regime (d). (e) Circuit simulation results and calculation of current flowing from ML through T2 as function of $V_{G,T1}$, in this case a saturation regime is considered. (f) Circuit simulation results and behavioral model of the inverter, with the model equation below. (g) Circuit simulations results and (h) model calculation of the overall discharge currant as function of V_{DL} and G_{Mi} assuming the same conductance programmed in both memristor of a analog CAM cell.

Constant	Value
I_{D0}	50 nA
α	80 mV
I'_{D0}	45 nA
k_1	160 μA/V
$V_{th,T1}$	405 mV
k_2	300 μA/V^2
$V_{th,T2}$	350 mV
β	50 V^{-1}
γ	-0.4 V

Supplementary Table 1. Analog CAM model fitting parameters

2 Supplementary Note 2: Extended figures of merit

In tree based algorithm acceleration the figure of merit are normalized to the node operation[2]. In fact, the basic operation that needs to be performed during inference is the evaluation of each threshold and decision upon which direction to go. This include a comparison and a memory lookup for the next comparison to make. For KUL Belgium Traffic Signs dataset classification we implemented a RF with 15 trees and a maximum depth of 10 which resulted in a $N_o = 4000$ nodes to be evaluated. Considering that each analog CAM cell can map $n_{bits} = 4$, we consider a 4 bit operation as the minimum operation size. We can compute the performance τ for the pipelined architecture with throughput f as

$$\tau = N_o \cdot f = 1.33 \ \ TOPS \tag{1}$$

and the energy efficiency η as

$$\eta = \frac{\tau}{Power} = 3.11 \ \ TOPS/W \tag{2}$$

Which corresponds to an improvement of 461$\times$ and 7.64$\times$ of the state of the art[2].

Note that this calculation consider the peak performance, which is reached for an optimal usage of the memory, with all the memory elements used at each cycle. This should be consider not as the algorithmic performance but as the hardware performance. In fact, considering the algorithm, a conventional non parallel processor given a tree with ν nodes, needs to perform $h = log_2\nu$ operations to traverse the tree and reach a leaf, which is typically referred as the height of a tree. By considering that, the total number of algorithmic operation N_1 that analog CAM performs at each cycle is

$$N_1 = N_{trees} \cdot log_2\nu = 15 \cdot log_2 256 = 120 \tag{3}$$

Thus the algorithmic performance can also be computed. For simplicity, hardware and algorithmic performance of analog CAM and SRAM based[2] accelerators are summarized in table 2

Accelerator	Type	Nodes	τ	η
aCAM	Hardware	4000	1.33 TOPS	3.11 TOPS/W
SRAM	Hardware	1984	2.89 GOPS	407 GOPS/W
aCAM	Algorithmic	120	33.960 GOPS	80.28 GOPS/W
SRAM	Algorithmic	317	231 MOPS	32.53 GOPS/W

Supplementary Table 2. Analog CAM and SRAM[2] accelerator performance

The calculation highlights that the main benefit of analog CAM accelerator is the massive parallel operation, and that should be considering during the mapping procedure.

3 Supplementary Note 3: Area comparison with SRAM accelerator

Previously[3], we optimized the cell layout of analog CAM at 16 nm technology along with the peripheral circuits. The resulting 6T2M analog CAM area is calculated to be $A_{6T2M} = 0.51 \mu m^2$. To perform RF inference, the state of each match line (ML) needs to be store in a register. The register area per bit at 16 nm is $A_{reg} = 5.5 \mu m^2$. After collecting all the ML results, the outputs need to be aggregated with a AND operation before performing the majority voting. Majority voting can be performed with traditional digital logic or with a matrix vector multiplication on a 1T1R RRAM array, as presented in the main text. Here for simplicity we consider to compute the majority voting with traditional digital logic. Every logic per bit has an area of $A_{logic} = 0.28 \mu m^2 \cdot 2 = 0.56 \mu m^2$ where the factor 2 is to consider the logic placement utilization due to routing overhead. The area the current steering digital to analog converter (Supplementary Figure 8) to be $A_{DAC} = 10 \cdot A.N_{ch} + 5 \cdot A_{mir}$ where $A_u = 0.046 \mu m^2$ is the area of a transistor with a single finger, $A_{mir} = 0.061 \mu m^2$ is the area of the current mirror transistors and N_{ch} the total number of desired channels.

Hence, the total area occupation of the analog CAM accelerator in 16 nm technology can be computed as:

$$A_{aCAM} = [N_{arrays} \cdot (H \cdot W \cdot A_{6T2M} + H \cdot A_{reg} + H \cdot A_{AND} + H \cdot A_{MAJ}) + A_{DAC}] \cdot (1 + OH) = 0.266 mm^2 \quad (4)$$

Where $N_{arrays} = 29$ is the total number of arrays in the architecture, $H = 480$ and $W = 16$ is the number of rows and columns in each array respectively, $N_{ch} = W \cdot N_a rrays$ for the full pipelined implementation, A_{AND} is the area of an AND gate, $A_{MAJ} = 3 \cdot A_{AND}$ the area of a majority voting gate, and $OH = 0.2$ is an extra overhead factor for placement and routing. With a fixed clock frequency of $f_{clk} = 1GHz$ the throughput at 16 nm remains the same of the previously calculated (Supplementary Information 2) at 65 nm, leading to the same performance τ. Thus the area efficiency α can be computed as

$$\alpha = \frac{\tau}{A_{aCAM}} = \frac{1.33 TOPS}{0.266 mm^2} = 5 \ \frac{TOPS}{mm^2} \quad (5)$$

To compare this result with the previously reported SRAM based accelerator[2] which is based on 65 nm technology, we need to scale it first to 16 nm. In TSMC process high density 6T SRAM bit cell has an area of $0.499 \mu m^2$ at 65 nm and $0.07344 \mu m^2$ at 16 nm. Hence the scaling factor is $0.499/0.07344 \sim 6.8$. Note however that in the reference, the bit cell area size is $2.11 \mu m \times 0.92 \mu m = 1.94 \mu m^2$ which is several times larger than the high density bit cell. This is due to up-sizing of the pass-gate and pull-down transistors to reduce the resistance of the bit line discharge path, which helps to increase the bit line voltage discharge swing. It can be expected that the 16 nm bit cell would need to be up-sized in a similar way, keeping the ratio of ~ 6.8. Thus we can estimate the area of the 6T SRAM bit cell at 16 nm as $A_{6T} = (1.94 \mu m^2)/6.8 = 0.285 \mu m^2$. Similarly, we can compute the scaling of the logic. Approximate metric for density scaling is the product of contacted poly pitch (CPP) and minimum metal pitch (MXP). In TSMC process $CPP \times MXP = 0.16 \times 0.18 \mu m^2$ at 65 nm and $CPP \times MXP = 0.09 \times 0.064 \mu m^2$ at 16 nm, leading to a scaling factor $(0.16 \times 0.18)/(0.09 \times 0.064) = 5$. Nevertheless, improvements in cell library design for advanced technology nodes can further improve this scaling ratio. For example, TSMC reports us to 2.35x density improvement from 65 to 40 nm, 2x improvements from 40 to 22 nm, and 1.44x improvements from 22nm to 16 nm, yielding to a cumulative logic density improvement of 6.8x from 65 to 16 nm. For considering a best-case scenario for designing the SRAM logic at 16 nm we consider a logic density scaling factor of 6.8x, which matches the scaling factor for the SRAM cell. We can directly measure the memory area (bit cell array) and logic (CTRL) from the layout[2], which at 65 nm results in a total of $A_{SRAM65} = 0.56 mm^2$. By applying the aforementioned scaling rules we can compute the area at 16 nm as $A_{SRAM16} = A_{SRAM65}/6.8 = 0.082 mm^2$ which leads to an area efficiency of

$$\alpha_{SRAM} = \frac{2.89 GOPS}{0.082 mm^2} = 35.24 \ \frac{GOPS}{mm^2} \quad (6)$$

While the area occupation for SRAM based accelerator is significantly lower compared to analog CAM accelerator, the resulting area efficiency is also drastically lower. This is due to the full parallelism that it is possible to achieve thanks to analog CAM. It can be thus concluded that the analog CAM accelerator is 142× more area efficient than the SRAM based accelerator[2].

Supplementary References

1. Li, C. *et al.* CMOS-integrated nanoscale memristive crossbars for CNN and optimization acceleration. In *2020 IEEE International Memory Workshop (IMW)*, 1–4, DOI: 10.1109/IMW48823.2020.9108112 (2020).
2. Kang, M., Gonugondla, S. K., Lim, S. & Shanbhag, N. R. A 19.4-nJ/Decision, 364-K Decisions/s, In-Memory Random Forest Multi-Class Inference Accelerator. *IEEE J. Solid-State Circuits* **53**, 2126–2135, DOI: 10.1109/JSSC.2018.2822703 (2018).
3. Li, C. *et al.* Analog content-addressable memories with memristors. *Nat. Commun.* **11**, 1638, DOI: 10.1038/s41467-020-15254-4 (2020).
